# Diverse Clinical Spectrum of TTN-Truncation Variant Cardiomyopathy: A Case Series Illustrating Asymptomatic, Arrhythmic, Recurrent Takotsubo, and Inflammatory Phenotypes

**DOI:** 10.7759/cureus.116868

**Published:** 2026-09-24

**Authors:** Mehran Jalilzadehbinazar, Craig K Reiss, Shane J LaRue, Mark A Gdowski, Anupama K Rao

**Affiliations:** 1 Cardiology, St. Luke's Hospital, Chesterfield, USA

**Keywords:** arrhythmia, dilated cardiomyopathy, genetic testing, takotsubo cardiomyopathy, titin, ttn

## Abstract

Truncating variants in the *TTN* gene (TTNtv) are the most common genetic causes of dilated cardiomyopathy (DCM). Despite this high prevalence, the clinical spectrum of TTNtv-associated cardiomyopathy remains incompletely characterized, with variable penetrance, expressivity, and phenotypic manifestations. In this study, we present four cases of TTNtv cardiomyopathy illustrating diverse clinical presentations: an asymptomatic phenotype with moderate left ventricular systolic dysfunction discovered through screening, a severe arrhythmic phenotype with ventricular tachycardia storm requiring multiple ablations and implantable cardioverter-defibrillator (ICD) shocks, a recurrent stress-induced (takotsubo) presentation with two discrete episodes, which we present as a hypothesis-generating observation, and an inflammatory presentation with fluorodeoxyglucose-positron emission tomography (FDG-PET) evidence of active myocardial inflammation and symptomatic response to corticosteroids. These cases illustrate the potential clinical value of genetic testing, cardiac magnetic resonance imaging, and individualized assessment in TTNtv cardiomyopathy, and suggest that genetic counseling and cascade testing should be considered for affected individuals and their families.

## Introduction

Dilated cardiomyopathy (DCM) is a common myocardial disorder characterized by left ventricular or biventricular dilatation and systolic dysfunction, affecting up to 1 in 250 individuals [1]. Among its genetic causes, truncating variants in the *TTN* gene (TTNtv) represent the most prevalent etiology, identified in approximately 25% of familial cases and 18% of sporadic cases [2]. TTN encodes titin, the largest human protein, which spans half the sarcomere, provides most of its passive force, and modulates active contraction [2]. TTNtv are also found in approximately 1-3% of the general population, often as incidental findings, with subclinical effects including eccentric remodeling and reduced left ventricular function [1,3,4]. The clinical significance of an individual TTNtv depends on factors including variant location, the proportion of cardiac transcripts that incorporate the affected exon, and the pathogenicity classification; not all incidentally identified TTNtv carry equivalent clinical risk [1].

The clinical manifestations of TTNtv cardiomyopathy are highly variable, ranging from asymptomatic individuals with preserved ejection fraction to those with end-stage heart failure requiring transplantation [2,5]. Arrhythmias, particularly atrial fibrillation and ventricular arrhythmias, are common and may precede the diagnosis of DCM [5-7]. Notably, TTNtv have been reported at increased frequency in patients with conditions traditionally considered acquired, including peripartum cardiomyopathy, alcohol-induced cardiomyopathy, and myocarditis [8-10]. The enrichment of TTNtv in patients with acute myocarditis and reduced left ventricular ejection fraction has led to a proposed "multiple hit" hypothesis, in which genetic susceptibility combined with an environmental trigger (e.g., viral infection, inflammation, pregnancy, or alcohol use) may unmask clinical disease [8,9,11]. This remains a proposed mechanism rather than an established causal pathway.

Cardiac magnetic resonance imaging (CMR) with late gadolinium enhancement (LGE) has emerged as an important tool for risk stratification in DCM, with specific LGE patterns associated with different genotypes [12]. Patients with TTNtv typically show nonspecific LGE patterns, but the presence of LGE, particularly when extensive, is a strong predictor of arrhythmic risk [6,12]. Genetic testing has become an integral component of DCM evaluation, enabling cascade screening of at-risk relatives and informing prognosis and management [4,11].

We present four cases of TTNtv cardiomyopathy that illustrate the phenotypic diversity of this condition, with implications for diagnosis, risk stratification, and treatment.

## Case presentation

Case 1: asymptomatic phenotype

A 72-year-old man with a history of anxiety and a family history of coronary artery disease and myotonic dystrophy underwent screening with a coronary artery calcium score. The score was elevated at 405 (86th percentile). A subsequent echocardiogram revealed moderate global left ventricular systolic dysfunction with a left ventricular ejection fraction (LVEF) of 37%. The patient was entirely asymptomatic (New York Heart Association Class I), denying exertional dyspnea, chest pain, or palpitations. Left heart catheterization revealed only mild non-obstructive coronary artery disease. CMR (Figure 1) confirmed non-ischemic cardiomyopathy with an LVEF of 37%, demonstrating a ring-like LGE pattern in the basal inferoseptum and subepicardial enhancement in the basal inferior and inferolateral walls.

**Figure 1:**
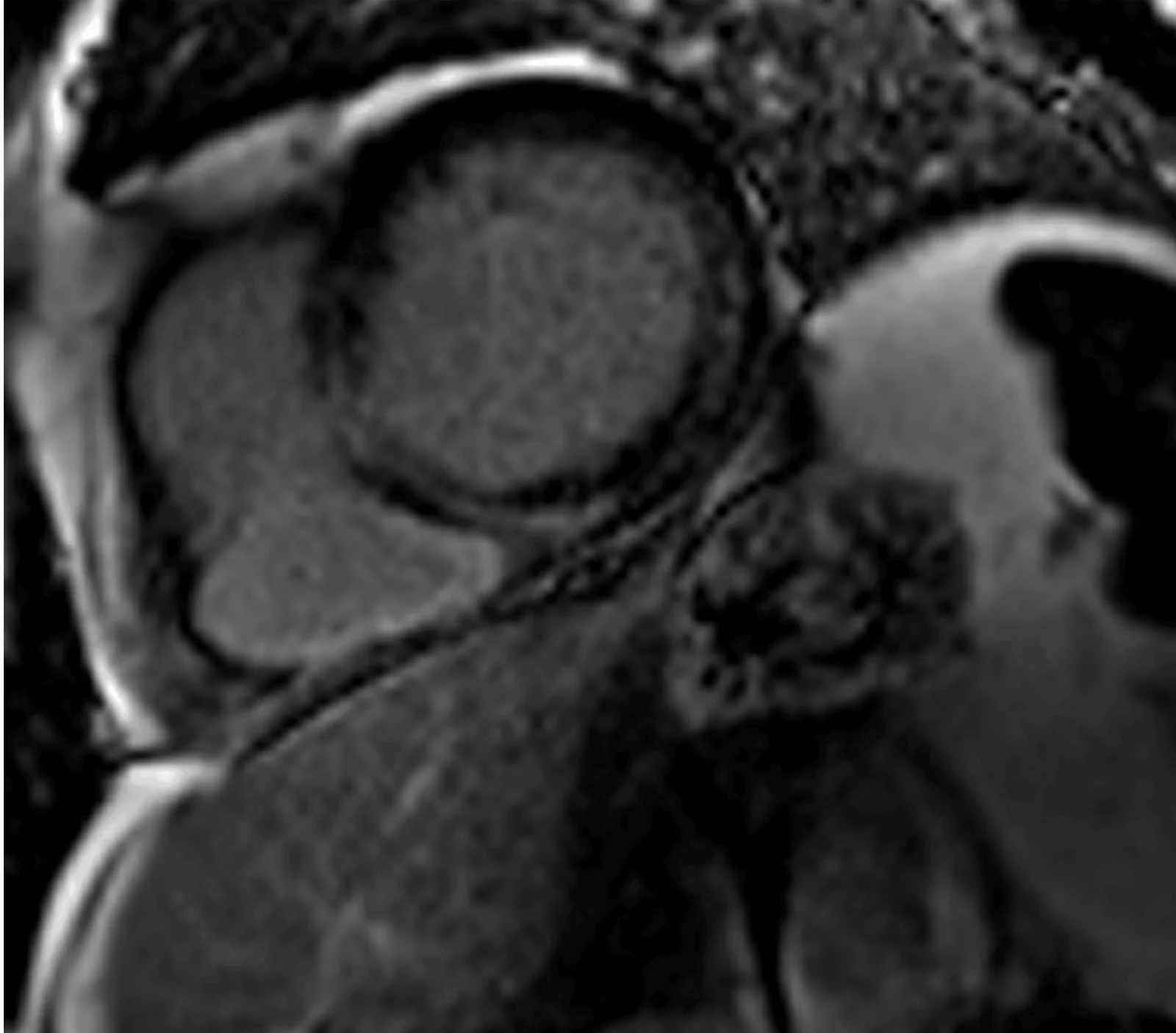
CMR late gadolinium enhancement image from Case 1 demonstrating ring-like LGE in the basal inferoseptum and subepicardial enhancement in the basal inferior and inferolateral walls, consistent with non-ischemic cardiomyopathy. CMR: cardiac magnetic resonance imaging; LGE: late gadolinium enhancement

T1 mapping demonstrated diffuse elevation in myocardial T1 values, suggesting the presence of myocardial scar or fibrosis, with normal T2 values indicating the absence of active inflammation (Figure 2).

**Figure 2:**
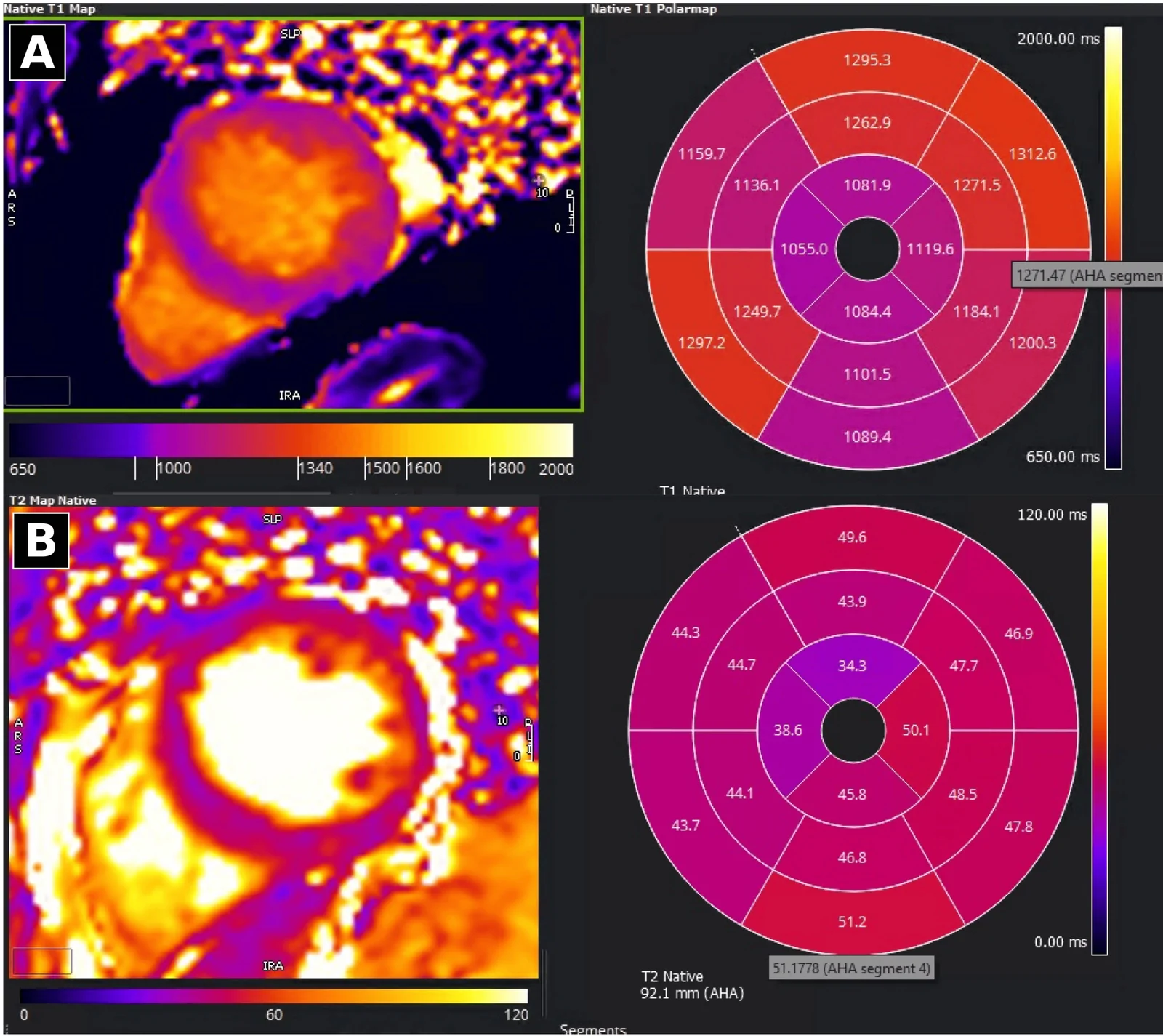
CMR T1 map (A) and T2 map (B). Diffuse elevation of T1 values is consistent with myocardial fibrosis. T2 values are within normal limits, arguing against active myocardial inflammation. CMR: cardiac magnetic resonance imaging

Given the non-ischemic etiology and family history of myotonic dystrophy, a condition also associated with cardiac involvement, genetic testing (Invitae; Labcorp Genetics, San Francisco, California, USA) was pursued and identified a heterozygous likely pathogenic TTN truncating variant. The patient was initiated on guideline-directed medical therapy (GDMT) and remains asymptomatic with well-controlled vitals on follow-up. Cascade genetic testing for first-degree relatives was recommended.

This case illustrates that TTNtv can be discovered incidentally in asymptomatic individuals and that genotype-positive individuals may remain phenotypically silent for decades.

Case 2: arrhythmic phenotype

A 67-year-old man with a long-standing history of non-ischemic cardiomyopathy, initially attributed to alcohol use, presented with a severe arrhythmic phenotype. His course was complicated by ventricular tachycardia (VT) storm requiring multiple shocks, transfers to a tertiary care center, and two catheter ablation procedures. His LVEF was severely reduced (~20%). Initial genetic testing was reported as negative; however, after his son was found to carry a pathogenic TTN variant, reanalysis of the patient's sample confirmed the same heterozygous TTN truncating variant.

Management has involved maximized GDMT, antiarrhythmic therapy with amiodarone and mexiletine, and cardiac resynchronization therapy defibrillator (CRT-D) implantation. Ongoing alcohol use is thought to contribute to a mixed TTNtv and alcohol-induced cardiomyopathy. CMR revealed extensive septal scar correlating with his arrhythmogenic substrate. LGE imaging demonstrated a wide-band subendocardial scar in the basal septum with a distribution resembling an ischemic (infarct-type) pattern (Figure 3). Obstructive coronary artery disease had been excluded, and the scar was therefore attributed to a non-ischemic cause in the setting of long-standing cardiomyopathy. LGE distribution alone does not establish etiology. He has been evaluated as a potential cardiac transplant candidate should VT become intractable.

**Figure 3:**
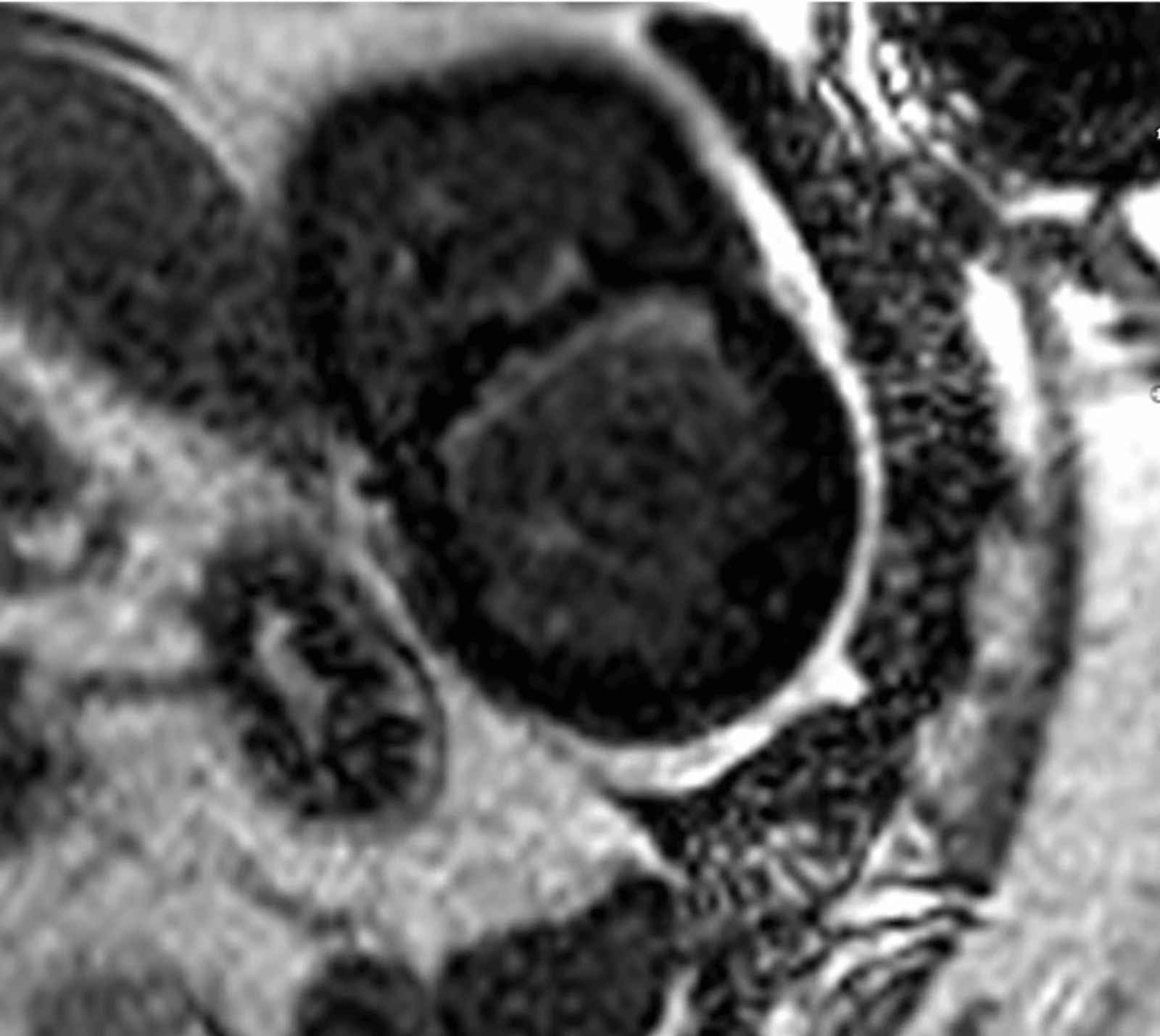
CMR with late gadolinium enhancement demonstrating wide-band subendocardial scar in the basal septum in a patient without obstructive coronary artery disease. CMR: cardiac magnetic resonance imaging

This case highlights the diagnostic challenges posed by concurrent environmental triggers and the severity of the arrhythmic phenotype in TTNtv cardiomyopathy.

Case 3: recurrent stress-induced (takotsubo) presentation

A 68-year-old woman with hypothyroidism presented with her second discrete episode of acute heart failure, the first having occurred three years earlier. Both presentations were characterized by acute substernal or back pain, hypoxia, and significantly reduced LVEF (25-33%) on left heart catheterization in the context of non-obstructive coronary artery disease. The wall motion pattern was consistent with takotsubo cardiomyopathy, with apical and mid-ventricular akinesis. LV function recovered substantially (to 50-60%) within weeks of each episode.

Following the second event, genetic testing was pursued given a family history of a deceased grandchild with suspected cardiomyopathy and a daughter with a known TTN variant. This revealed a heterozygous pathogenic TTN variant (c.64094-2A>G). A surveillance CMR obtained after recovery showed normalized biventricular function but subtle midwall LGE (Figure 4) and globally elevated T1/T2 values suggestive of residual edema or low-level inflammation (Figure 5).

**Figure 4:**
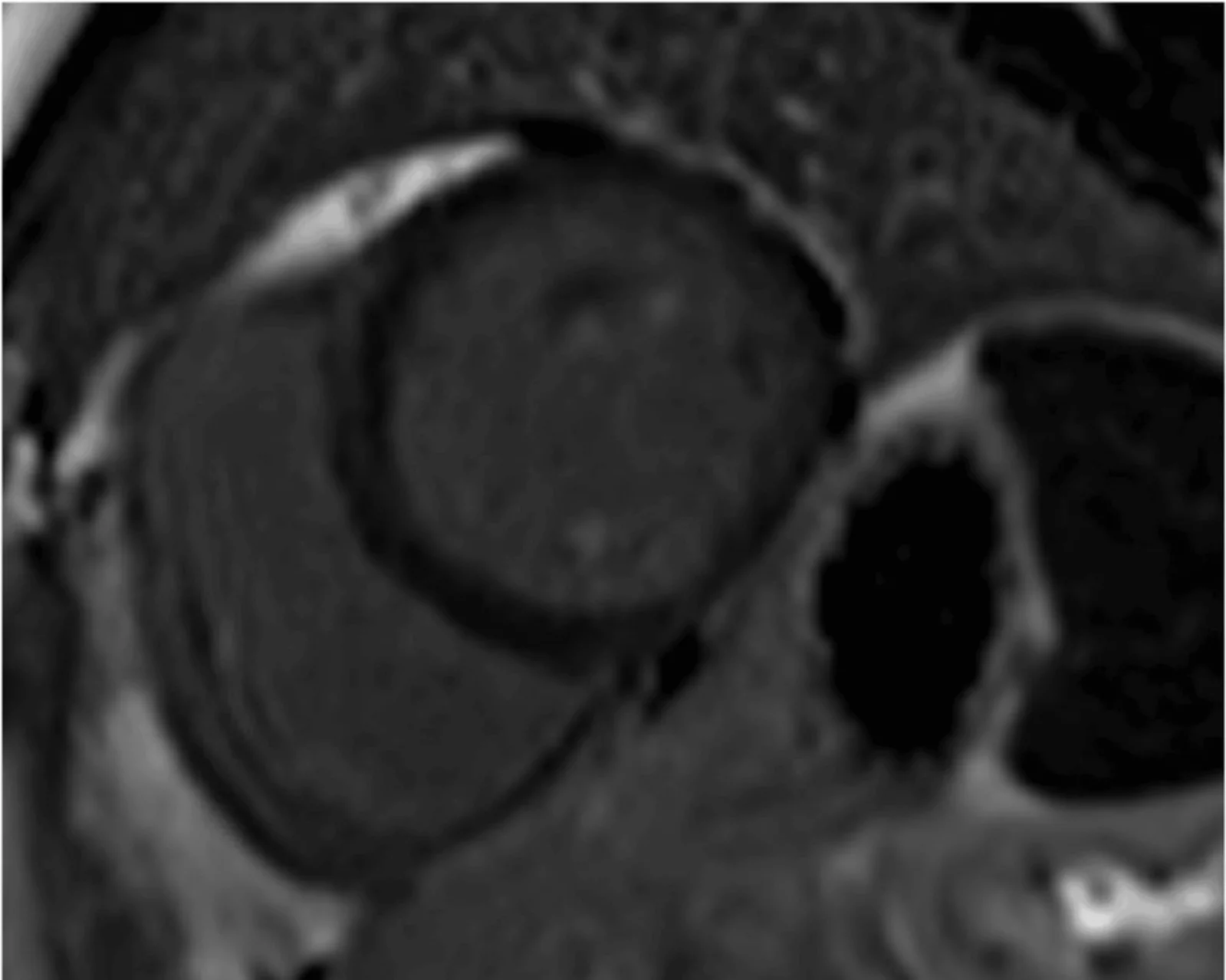
CMR with late gadolinium enhancement image demonstrating subtle midwall LGE obtained after recovery from the second takotsubo episode. CMR: cardiac magnetic resonance imaging; LGE: late gadolinium enhancement

**Figure 5:**
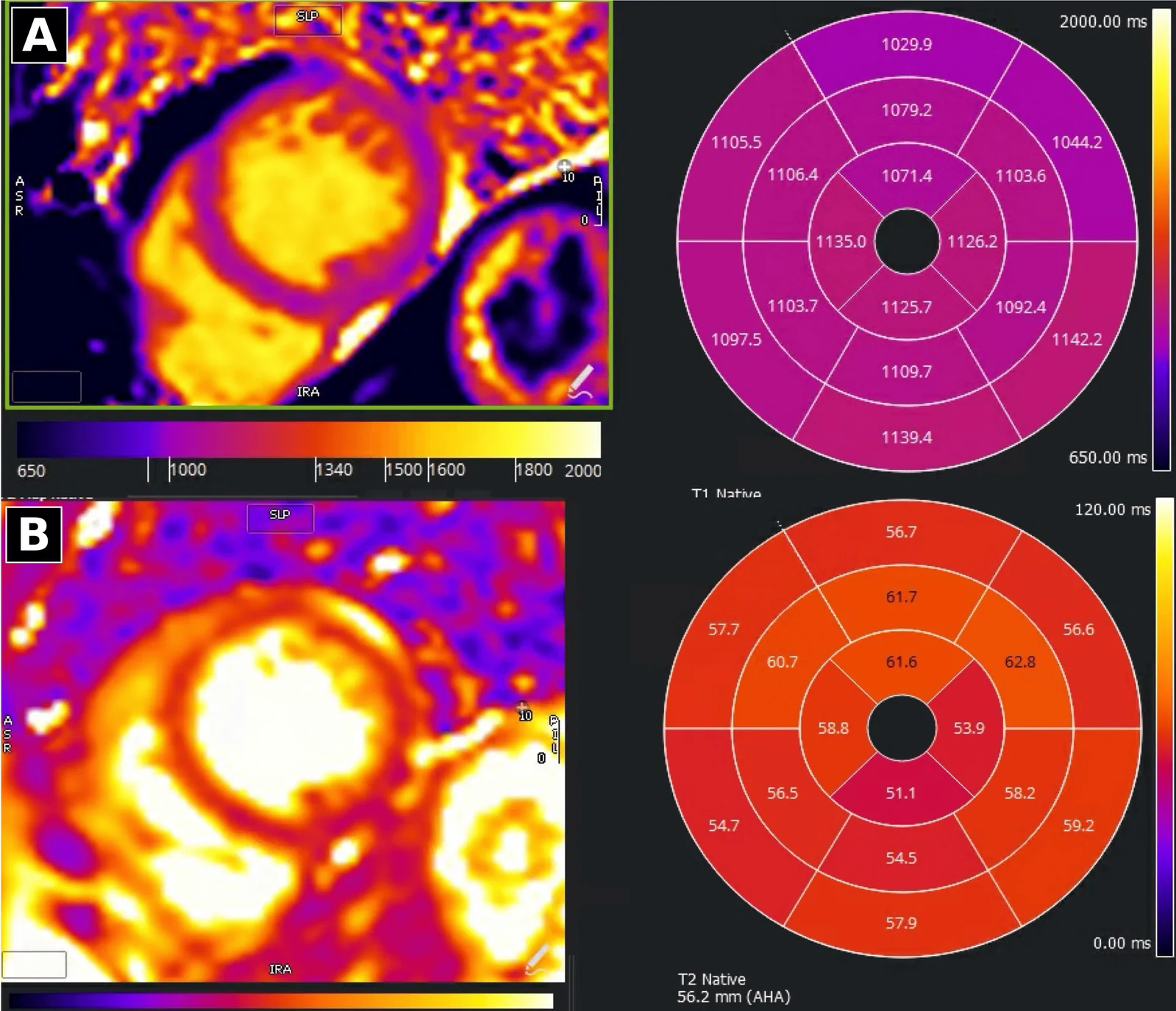
CMR T1 map (A) and T2 map (B) demonstrating globally elevated T1 and T2 values, consistent with residual myocardial edema or low-level inflammation following the takotsubo episode. CMR: cardiac magnetic resonance imaging

Whether TTNtv lowers the threshold for recurrent stress-induced (takotsubo) cardiomyopathy cannot be determined from a single case. This observation is hypothesis-generating and would require systematic study.

Case 4: inflammatory phenotype

A 44-year-old athletic man presented with acute chest pain and dyspnea three months after COVID-19 vaccination. Vaccination is a recognized risk factor for myocarditis [11]; in this case, however, the relationship was a temporal association only, and no causal link was established. Initial workup, including a CMR, was unrevealing. Over the ensuing years, he developed persistent symptoms and arrhythmias, including paroxysmal supraventricular tachycardia and premature ventricular complexes. Genetic testing, prompted by his brother's diagnosis of TTNtv DCM, revealed the same likely pathogenic TTN truncating variant (c.96206T>A, p.Leu32069).

Serial surveillance CMRs documented progression from a normal baseline study to one showing reduced LVEF (49-50%) and subepicardial LGE in the basal inferolateral wall (Figure 6), with corresponding T1 and T2 mapping demonstrating subepicardial signal elevation and elevated myocardial T1 and T2 values suggestive of concomitant inflammation and fibrosis (Figure 7). When symptoms of fatigue and exertional dyspnea worsened significantly in 2025, a fluorodeoxyglucose-positron emission tomography (FDG-PET) scan was obtained and demonstrated patchy, avid FDG uptake colocalizing with the LGE regions, consistent with active myocardial inflammation (Figure 8). Notably, standard inflammatory markers (C-reactive protein (CRP), erythrocyte sedimentation rate (ESR)) and N-terminal pro-B-type natriuretic peptide (NT-proBNP) were within normal limits. A trial of GDMT provided no symptomatic relief. A therapeutic challenge with corticosteroids was followed by marked symptomatic improvement within 24 hours; symptoms recurred upon tapering and improved again with re-initiation of corticosteroids.

**Figure 6:**
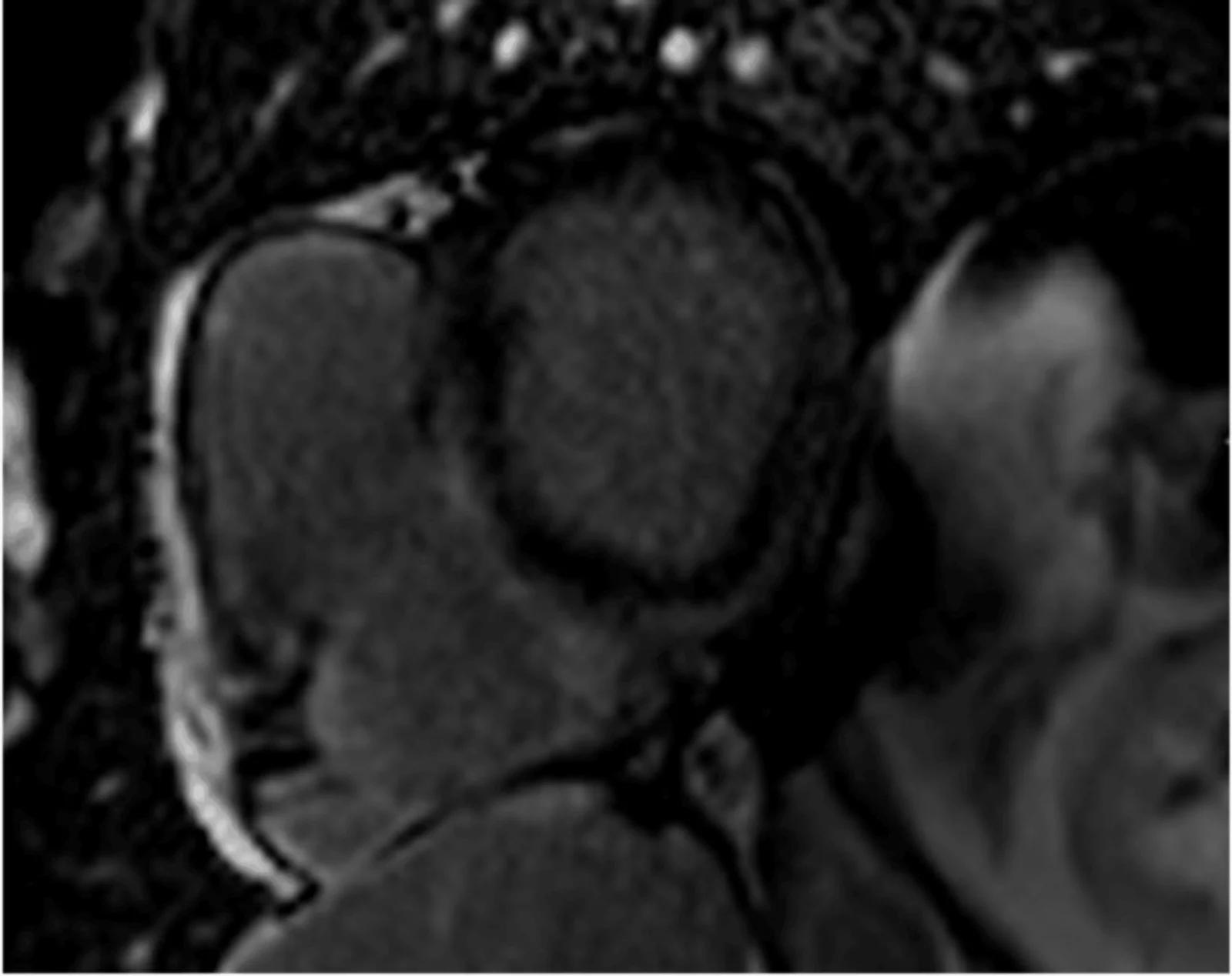
CMR with late gadolinium enhancement image demonstrating subepicardial LGE in the basal inferolateral wall. CMR: cardiac magnetic resonance imaging; LGE: late gadolinium enhancement

**Figure 7:**
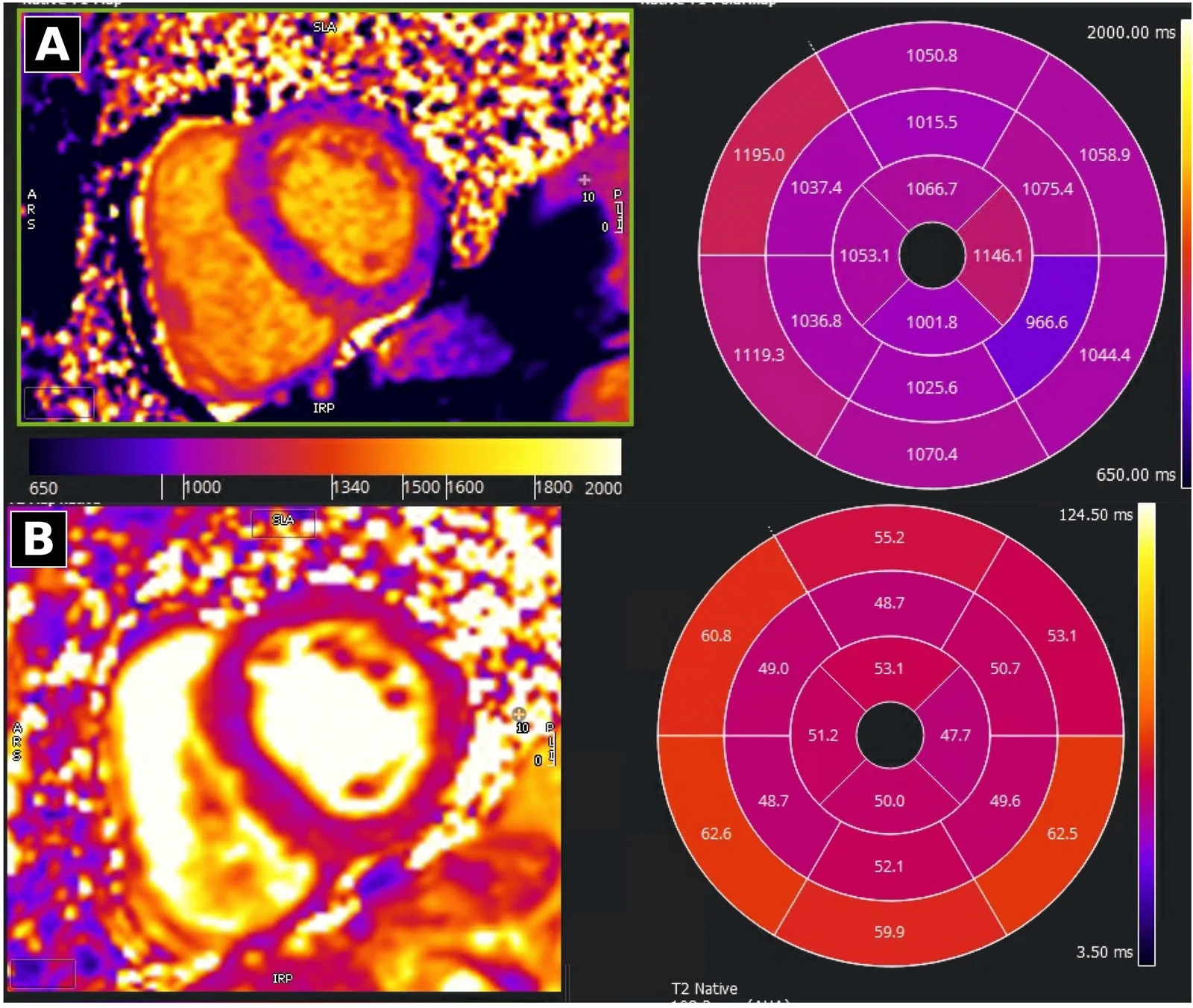
CMR T1 map (A) and T2 map (B) showing subepicardial signal elevation with elevated myocardial T1 and T2 values, consistent with active myocardial inflammation superimposed on fibrosis. CMR: cardiac magnetic resonance imaging

**Figure 8:**
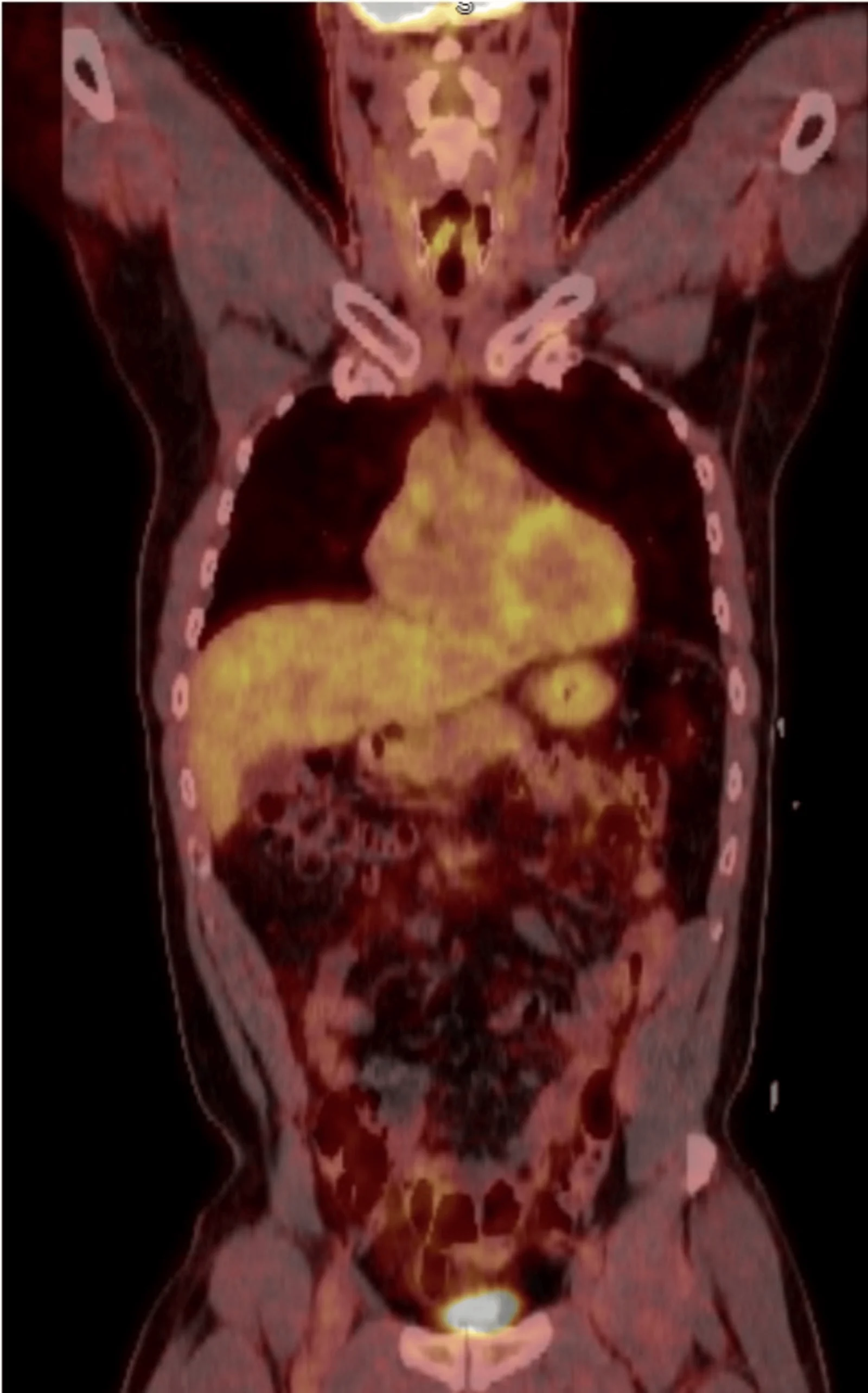
FDG-PET study demonstrating patchy, avid myocardial FDG uptake colocalizing with the regions of LGE identified on CMR, consistent with active myocardial inflammation. FDG-PET: fluorodeoxyglucose-positron emission tomography; CMR: cardiac magnetic resonance imaging; LGE: late gadolinium enhancement

This case describes a genetically confirmed TTNtv carrier with imaging evidence of active myocardial inflammation whose symptoms did not improve on GDMT but improved after corticosteroids. As a single uncontrolled observation, it cannot establish the comparative efficacy of immunosuppression. 

The clinical, genetic, imaging, treatment, and outcome data for all four cases are summarized in Table 1.

**Table 1:** Summary of clinical, genetic, imaging, treatment, and outcome data CAD: coronary artery disease; CMR: cardiac magnetic resonance; CRT-D: cardiac resynchronization therapy defibrillator; GDMT: guideline-directed medical therapy; LGE: late gadolinium enhancement; LVEF: left ventricular ejection fraction; N/A: not available; PVC: premature ventricular complex; SVT: supraventricular tachycardia; VT: ventricular tachycardia; ICD: implantable cardioverter-defibrillator; FDG-PET: fluorodeoxyglucose positron emission tomography; DCM: dilated cardiomyopathy; LV: left ventricular

	Case 1	Case 2	Case 3	Case 4
Age, sex	72, male	67, male	68, female	44, male
Presentation	Asymptomatic; elevated coronary artery calcium score (405) prompted echocardiography	VT storm with ICD shocks on a background of long-standing non-ischemic cardiomyopathy attributed to alcohol	Second episode of acute heart failure with chest or back pain and hypoxia	Acute chest pain and dyspnea; later persistent symptoms and arrhythmias
LVEF	37%	~20%	25-33% acutely; 50-60% after recovery	49-50% (normal at baseline)
Coronary evaluation	Mild non-obstructive CAD on catheterization	Obstructive CAD excluded	Non-obstructive CAD on catheterization	N/A
TTN variant	Truncating; likely pathogenic	Truncating; pathogenic	c.64094-2A>G; pathogenic	c.96206T>A, p.Leu32069; likely pathogenic
Reason for genetic testing	Non-ischemic etiology; family history of myotonic dystrophy	Son found to carry pathogenic TTN variant; sample reanalyzed	Daughter with known TTN variant; deceased grandchild with suspected cardiomyopathy	Brother diagnosed with TTNtv DCM
CMR LGE	Ring-like basal inferoseptal; subepicardial basal inferior and inferolateral	Wide-band subendocardial basal septal	Subtle midwall (after recovery)	Subepicardial basal inferolateral
T1/T2 mapping	Diffusely elevated T1; normal T2	N/A	Globally elevated T1 and T2	Elevated T1 and T2
FDG-PET	Not performed	Not performed	Not performed	Patchy avid uptake colocalizing with LGE
Arrhythmias	None reported	VT storm; two catheter ablations	None reported	Paroxysmal SVT; PVCs
Treatment	GDMT	GDMT; amiodarone; mexiletine; CRT-D; transplant evaluation	N/A	GDMT without symptomatic response; corticosteroids with response
Status at last follow-up	Asymptomatic on GDMT	Ongoing VT management	Recovered LV function	Corticosteroid-responsive symptoms

## Discussion

This case series demonstrates the phenotypic heterogeneity of TTNtv cardiomyopathy and illustrates the potential value of genetic testing in patients with cardiomyopathy of uncertain etiology [2,4,11].

TTNtv as the most common genetic cause of DCM

Herman et al. established that TTN truncating mutations are the most common genetic cause of DCM, occurring in approximately 25% of familial cases and 18% of sporadic cases, with greater than 95% observed penetrance after the age of 40 years [2]. Mutations are overrepresented in the titin A-band, a skewed distribution that Herman et al. interpreted as favoring a dominant-negative effect over pure haploinsufficiency, though the precise pathophysiology remains an area of active investigation [2]. The presence of TTNtv in the general population, with a weaker association with DCM in individuals of African ancestry, highlights the complex interplay between genetic susceptibility and environmental factors [3]. In an observational analysis, early initiation of beta-adrenergic receptor or RAS-blocking therapy in asymptomatic TTNtv-positive individuals was associated with lower odds of DCM onset [10]. Prophylactic treatment of genotype-positive, phenotype-negative relatives identified through cascade screening is therefore an emerging strategy, but it is not an established standard of care and has not been evaluated in randomized trials.

Arrhythmic risk and risk stratification

Corden et al. demonstrated that TTNtv carriers with implanted defibrillators had a significantly increased risk of appropriate ICD therapy, with the combination of TTNtv and midwall fibrosis conferring a 16.6-fold increased risk compared with patients without either risk factor [6]. Verdonschot et al. further showed that TTNtv patients with an additional environmental trigger had particularly prominent arrhythmias, an observation consistent with the proposed "multiple hit" hypothesis [7]. The arrhythmic burden in TTNtv cardiomyopathy may be driven by altered mitochondrial energetics, increased interstitial fibrosis, and reduced cardiac mass [7]. Notably, TTNtv are not classified among high-risk arrhythmic genes such as PKP2, which carry higher arrhythmic risk even in the absence of a DCM phenotype [13]; however, the presence of extensive LGE, as observed in Case 2, substantially modifies the risk profile.

Inflammatory overlap and gene-environment interactions

TTNtv are enriched in patients with myocarditis and reduced LVEF, supporting a shared genetic predisposition between inflammatory cardiomyopathy and DCM [8,9,11]. Lota et al. found that TTNtv were present in 7% of myocarditis patients with LVEF below 50% compared with 1% of controls, with genotype-positive patients demonstrating a numerically higher five-year mortality (11.1% vs. 3.3%), though this did not reach statistical significance after adjustment [8]. A meta-analysis by Monda et al. demonstrated that pathogenic variants in cardiomyopathy-associated genes are present in 21.9% of adults with complicated myocarditis, with TTN being the most commonly implicated gene [9]. The concept that TTNtv carriers may remain phenotypically silent until an environmental trigger unmasks the disease is the basis of the proposed "multiple hit" hypothesis [1,7,8,10,11]. Case 4 is compatible with this hypothesis but cannot demonstrate it. Peretto et al. summarized the emerging interplay between genetic susceptibility and myocardial inflammation, highlighting gene-immune interactions as key areas for future investigation [14]. The symptomatic response to corticosteroids in Case 4 is of interest in light of the TIMIC trial, in which immunosuppressive therapy in endomyocardial-biopsy-proven, virus-negative inflammatory cardiomyopathy was associated with persistent improvement in LVEF and better long-term outcomes [15]. However, the diagnosis in Case 4 rested on imaging, and a single uncontrolled symptomatic response cannot be equated with trial-level evidence; the diagnostic and therapeutic contexts differ.

Sex differences and clinical outcomes

Sex differences are clinically relevant in TTNtv cardiomyopathy. Herman et al. found that adverse events occurred earlier in male mutation carriers than in female carriers (mean age 56 vs. 68 years) [2]. Vissing et al. showed that female sex independently predicted lower rates of ventricular arrhythmias and was positively associated with left ventricular reverse remodeling (LVRR), occurring in 72% of women compared with 51% of men [5]. These differences may account in part for the more severe arrhythmic presentation in Case 2 (male) versus the stress-induced presentation in Case 3 (female), although this cannot be established from two cases.

Role of cardiac magnetic resonance imaging

CMR with LGE is an important tool for risk stratification in DCM. de Frutos et al. demonstrated that LGE patterns have genotype-specific distributions, with TTNtv patients showing nonspecific patterns [12]. The presence of LGE, particularly when extensive, is a strong predictor of arrhythmic risk [6,12]. Subepicardial LGE patterns, associated with DSP and FLNC variants, had the highest yield for genetic testing (52.9%), while LGE patterns in TTNtv patients are more varied, reflecting the condition's heterogeneity [12]. Elevated T1/T2 values in Cases 3 and 4 suggest residual edema or active inflammation, which may represent markers of disease activity, though their role in guiding therapy remains to be established [11].

Management and therapeutic implications

GDMT for heart failure with reduced ejection fraction now includes four medication classes (beta-blockers, ACEi/ARB/ARNi, MRAs, and SGLT2 inhibitors) per the 2022 AHA/ACC/HFSA guidelines [16]. ICD therapy is recommended for primary prevention in patients with LVEF of 35% or less and NYHA Class II-III symptoms [16]. In TTNtv-positive individuals who had not yet developed overt DCM, prophylactic beta-adrenergic receptor or renin-angiotensin system-blocking therapy was associated with an 87% reduction in the odds of DCM onset (odds ratio (OR): 0.13; 95% confidence interval (CI): 0.08-0.23) [10]. This is an observational association and does not establish treatment efficacy; whether pre-emptive pharmacological intervention benefits at-risk relatives requires prospective evaluation. In selected patients with confirmed active myocardial inflammation, after exclusion of active infection and ideally with endomyocardial biopsy confirmation, immunosuppressive therapy may be considered in line with consensus guidance [11,15]. Genetic testing should be considered in all patients with DCM, particularly those with familial disease, early onset, or atypical features, and cascade testing of at-risk relatives supports early detection and intervention [2,4,11].

Study limitations

This series has several limitations. It is a retrospective description of four cases, which limits generalizability. Not all patients underwent the same diagnostic workup, as some investigations were guided by clinical presentation rather than a standardized protocol. Complete variant nomenclature, quantitative mapping values, and formal arrhythmia burden data were not uniformly available across cases. Long-term follow-up is also incomplete for some cases. The associations proposed for the stress-induced and inflammatory presentations are hypothesis-generating and cannot be established from single cases. Despite these limitations, the series illustrates the phenotypic diversity of TTNtv cardiomyopathy and highlights clinical lessons relevant to practicing clinicians.

## Conclusions

TTNtv are the most common genetic causes of DCM, with a phenotypic spectrum that includes asymptomatic and arrhythmic presentations and, as observed in this series, stress-induced and inflammatory presentations; the latter two associations are hypothesis-generating. The phenotypic expression may be influenced by sex, environmental triggers, and the presence of myocardial fibrosis. Cardiac magnetic resonance imaging with LGE assessment is an important tool for risk stratification, and the combination of TTNtv with midwall fibrosis has been associated with particularly high arrhythmic risk. Genetic testing should be considered in patients with DCM and in those with myocarditis, enabling cascade screening of at-risk relatives and informing prognosis and management. In appropriately selected patients with confirmed inflammatory cardiomyopathy, immunosuppressive therapy may be considered; the present series cannot establish therapeutic efficacy. GDMT remains the cornerstone of management, with device therapy reserved for those at high arrhythmic risk. Future research should focus on identifying predictors of phenotypic expression, developing genotype-specific risk stratification tools, and evaluating targeted therapies for TTNtv cardiomyopathy.
